# Impact of different occlusal schemes on temporomandibular joint health among edentulous patients in India

**DOI:** 10.6026/973206300222168

**Published:** 2026-04-30

**Authors:** Ayushi Rajesh Gupta, Prashanth Shetty, Sayali Prakash Belkhode, Sandhya James, Sayan Pal

**Affiliations:** 1 Department of Prosthodontics, Triveni Institute of Dental Sciences, Hospital and Research Centre, Bilaspur, Chhattisgarh, India

**Keywords:** Temporomandibular joint (TMJ), occlusal scheme, edentulous patients, complete dentures, lingualized occlusion, bilateral balanced occlusion

## Abstract

Complete denture occlusal schemes impact Temporomandibular Joint (TMJ) health in edentulous patients, yet the comparative effects of bilateral balanced, lingualized and monoplane occlusion remain insufficiently defined. Hence, this prospective RCT randomized 75 fully edentulous patients into three groups (n=25 each) who received dentures with bilateral balanced, lingualized or monoplane occlusion and were evaluated at baseline, 3 and 6 months. Lingualized occlusion yielded the lowest Helkimo dysfunction scores, optimal condylar positioning on CBCT and minimal TMJ clicking/deviation compared with other schemes at 6 months. All groups improved maximum mouth opening, but lingualized occlusion showed statistically superior gains alongside the lowest VAS pain scores. Thus, lingualized occlusion is the optimal TMJ-preserving scheme for edentulous patients, guiding clinical denture fabrication.

## Background:

Total edentulism is not only a common occurrence in most parts of the world, especially in the geriatric population, but is also a major challenge to oral rehabilitation [1]. It is the loss of all natural teeth that essentially changes the biomechanics of the stomatognathic system, thereby influencing masticatory capacity, facial appearance, phonology and most significantly, the temporomandibular joint complex [2]. The temporomandibular joint is the most complicated type of human joint, which is especially vulnerable to functional disruptions in case the occlusal relationships have been affected by the loss of teeth and their consequent restoration with the help of prosthetic means [3]. Edentulism in relation to temporomandibular disorders is a controversial topic in the existing literature on prosthodontics. Several epidemiological studies have reported higher rates of TMJ signs and symptoms among complete denture wearers than among the dentate population, with reported prevalence rates ranging from 16 to 75%, depending on diagnostic criteria and study sample [4]. TMJ dysfunction etiology among patients who wear edentulous dentures is multifactorial and includes alterations in the vertical dimension of occlusion, changes in posterior tooth support, changes in condylar position and the natural instability of complete denture prostheses [5]. One of the most important factors in the success of the prosthesis and the patient's comfort is the occlusal scheme chosen to fabricate the complete denture. Three main concepts of occlusion have been popular in prosthodontic practice: bilateral balanced occlusion, lingualized occlusion and monoplane (zero-degree) occlusion [6]. The concept of bilateral balanced occlusion was first introduced in the early twentieth century to bring all teeth into contact during eccentric mandibular movements, thereby increasing denture stability and evenly distributing the forces of occlusion throughout the remaining ridges [7]. An easier-to-use form, lingualized occlusion, has the same effect as anatomic teeth on central fossae of mandibular teeth, with the benefits of esthetic appearance of the anatomic tooth applied to the mechanical benefits of reduced lateral forces [8]. Monoplane occlusion involves the use of non-anatomic flat teeth that are placed without a compensating curve to reduce destabilizing horizontal forces on the denture bases [9]. Although much of the research has investigated the effects of these occlusal schemes on masticatory efficiency, patient satisfaction and residual ridge resorption, little has been done to understand their direct effects on temporomandibular joint health [10].

The biomechanical explanation implies that varying occlusal designs would produce varying patterns of force transmission through the mandible to the TMJ complex, which may affect condylar loading, disc positioning and the development of joint pathology [11]. Recent studies employing advanced imaging techniques, such as cone-beam computed tomography, have facilitated more accurate assessment of condylar morphology and position within the glenoid fossa, providing objective parameters for evaluating TMJ health in prosthetically reconstructed patients [12]. Natural experiments that utilise electromyographic measurements have indicated that the choice of occlusal scheme has a pronounced impact on the patterns of masticatory muscle activity and this subsequently impacts the forces conveyed to the temporomandibular joint [13]. In addition, studies on jaw kinetics have shown that various occlusal positions elicit quantifiable variations in mandibular motion during functional activities [14]. Although these have been conducted, there remains a significant research gap in the direct comparative analysis of the effects of the bilateral balanced, lingualized and monoplane occlusal schemes on TMJ health outcomes in clinically meaningful follow-ups. The evidence in the literature has been based on two concepts of the occlusal, or has used a few assessment parameters, which have been unable to provide a broad view of the health status of the TMJ [15]. Also, a significant portion of the evidence is based on cross-sectional studies, which cannot determine temporal dependencies between the selection of occlusal schemes and the emergence of TMJ dysfunction [16]. Therefore, it is of interest to compare and assess the effects of three various occlusal schemes. Namely, bilateral balanced occlusion, lingualized occlusion and monoplane occlusion on the health of the temporomandibular joint in completely edentulous patients.

## Materials and Methods:

## Design and ethical considerations of the study:

It was a prospective, randomized and comparative clinical study conducted at the Department of Prosthodontics over a period of 18 months. Informed consent was signed by all participants after they had been well informed about the purpose of the study, the procedures involved, the risks that might arise and their right to withdraw without facing any consequences. The principles of the Declaration of Helsinki on medical research on human subjects were followed in the study protocol.

## Sample size determination:

The sample size was estimated using G*Power, with a projected medium effect size (f = 0.35), an alpha of 0.05 and a statistical power of 80. The formula indicated that at least 22 subjects were required per group. To account for the likelihood of dropouts, twenty-five cases were recruited in each group, yielding a total of 75 patients.

## Participant selection:

The outpatient clinic was used to recruit 75 patients who were completely edentulous and aged 45-70 years.

## Inclusion criteria:

Any complete edentulism at least six months, Atwood Class III or IV residual ridges in both arches, sufficient inter arch space to allow a prosthetic rehabilitation, no prior history of TMJ surgery or treatment and the desire to be followed up. The exclusion criteria were: the presence of maxillofacial trauma, the presence of diagnosed temporomandibular disorders that active treatment was required, systemic diseases that interfere with bone metabolism (e.g., rheumatoid arthritis, osteoporosis or Paget disease), neuromuscular or neurological conditions, severe residual ridge atrophy (Atwood Class V or VI), radiation treatment of the area of the head and neck and individuals who had already received a complete denture with satisfactory prosthesis.

## Group allocation and randomization:

A computer-generated randomization sequence was used to assign patients to three groups at random with sealed opaque envelopes:

[1] Group A: Studies on bilateral balanced occlusion are only reported in (n = 25).

[2] Group B: Lingualized occlusion (n = 25).

[3] Group C: Monoplane occlusion (n = 25).

## Total denture construction:

Any complete denture was manufactured under the standardized clinical and laboratory conditions. Initial impressions were created with an irreversible hydrocolloid substance on stock trays, then tailor-made trays were created with autopolymerizing acrylic resin, with a 2mm periphery around the vestibular reflection. Zinc oxide eugenol impression paste was used to obtain final impressions in border-molded custom trays. Jaw relation records were obtained based on the split-cast method of accuracy checks. The phonetic method and the physiologic rest position procedure (subtracting 23 mm of freeway space) were used in defining the vertical dimension of occlusion. The transfer of the faces was performed using an arbitrary face-bow and the records were placed on a semi-adjustable articulator. In Group A, the anatomic teeth (33-degree cusp angle) were set according to the Hanau quint principles, with a compensating curve, so that bilateral balanced contacts were found in protrusion and lateral excursions. In Group B, the anatomic teeth were utilized in the maxillary arch so that lingual cusps would be in contact with the central fossae of semi-anatomic mandibular teeth. In the case of Group C, zero-degree non- anatomic teeth were set on a flat occlusal plane without compensating curves. All dentures were fabricated using heat-polymerized polymethylmethacrylate resin, which has a long curing cycle. Remounting and selective grinding in the laboratory were performed to remove processing-induced occlusal discrepancies. Denture insertion was performed with border extension, occlusal contacts and adjustments to patient comfort. Every patient received the same post-insertion instructions and follow-up procedures were scheduled at 24 hours, 1 week and 2 weeks after insertion.

## Parameters and instruments of assessment:

The following parameters were used to assess TMJ health at three time points, including baseline (at the time of denture insertion, T0), three months (T1) and six months (T2) after denture insertion.

## Helkimo clinical dysfunction index (DI):

This validated index measures 5 clinical variables, including limitation of mandibular range of motion, impaired TMJ function, muscle pain on palpation, TMJ pain on palpation and pain on mandibular movement. The variables were rated out of 5 and the total score placed the patient in one of four groups: Di-0 (0 points, no dysfunction), Di-I (1-4 points, mild dysfunction), Di-II (5-9 points, moderate dysfunction) and Di-III (10-25 points, severe dysfunction).

## Visual analog scale (VAS) of TMJ Pain:

The patients were asked to rate their TMJ pain intensity on a horizontal VAS with 100mm, where 0 represents no pain and 100 represents the worst pain imaginable.

## Maximum mouth opening (MMO):

It is the maximum interincisal distance, measured in millimeters with 0.01 mm accuracy using a digital caliper. Measures were taken and averaged three times.

## Joint sound assessment:

Bilateral TMJ sounds (clicking, crepitus, or absent) were assessed with a Littmann Classic III stethoscope, during opening, closing and lateral excursive movement.

## Cone beam computed tomography (CBCT) analysis:

CBCT scans were obtained at baseline and at 6 months using a standardized protocol (field of view: 8 x 8 cm, voxel size: 0.2 mm, exposure parameters: 90 kVp, 10 mA). The location of the condylar in the glenoid fossa was measured using the anterior joint space (AJS), the superior joint space (SJS) and the posterior joint space (PJS) of the corrected sagittal sections at the centre of each condyle. The calculation of the condylar concentricity was made according to the formula: (P -A)/(P + A)100, where P and A are the anterior and posterior joint space, respectively. A zero value is indicative of perfect concentricity; positive values represent anterior positioning and negative values represent posterior positioning.

## Statistical analysis:

The statistical analysis of the data was carried out using SPSS software (version 26.0, IBM Corporation). The Shapiro-Wilk test was used to test the normality of data distribution. Continuous variables were reported as the mean and standard deviation. Intergroup comparisons were conducted using one-way analysis of variance (ANOVA), followed by pairwise comparisons using Tukey's post hoc test in case significant differences were observed. Intragroup comparisons across time points were performed using repeated-measures ANOVA. Categorical data were analyzed using the chi-square or Fisher's exact test. The statistical significance level was set at p < 0.05.

## Results:

At baseline (T0), there was no statistically significant difference among the three groups (BBO, LO, MO) for both Helkimo Score (p=0.891) and VAS pain (p=0.924), indicating comparable initial clinical status. At 3 months (T1), a significant reduction in both Helkimo score (p=0.018) and VAS pain (p=0.002) was observed among the groups. Group B (LO) showed the greatest improvement, followed by Group A (BBO), while Group C (MO) demonstrated the least improvement. At 6 months (T2), the differences became highly statistically significant for both Helkimo score (p=0.001) and VAS pain (p<0.001). Group B (LO) continued to show the maximum reduction in symptoms, indicating superior efficacy, whereas Group C (MO) showed comparatively minimal improvement. Overall, LO therapy demonstrated the best clinical outcomes, followed by BBO, with MO showing the least effectiveness over time (Table 1 - see PDF). Maximum mouth opening improved across all three groups over the study period, with statistically significant intragroup increases from baseline to six months in all groups (p < 0.05). However, Group B exhibited significantly greater improvement at six months compared to Group C (p = 0.012). The incidence of TMJ clicking sounds was highest in Group C at six months (33.3%), followed by Group A (16.7%) and Group B (8.3%), with the overall difference being statistically significant (p = 0.041) (Table 2 - see PDF). Radiographic assessment of condylar position revealed important differences among groups at the six-month follow-up. The condylar concentricity values in Group B were closest to zero, indicating the most concentric condylar position within the glenoid fossa. Group C demonstrated the greatest deviation from concentricity, with condyles tending toward a more posterior position. The anterior and posterior joint space measurements showed significant intergroup differences at six months (Table 3 - see PDF).

## Discussion:

The results of the study indicate that the choice of occlusal scheme used in the fabrication of a complete denture has a quantifiable, clinically significant effect on temporomandibular joint health in edentulous patients. The lingualized occlusion condition had all evaluated TMJ health parameters in a better state than both the bilateral balanced and the monoplane occlusion conditions throughout the six-month observation period. It is possible to explain the better outcome of lingualized occlusion to preserve the health of the TMJ because of its peculiar biomechanical features. This scheme can greatly reduce the number of lateral force vectors generated by directing occlusal forces to relatively flat, wide mandibular occlusal surfaces, primarily through the lingual cusps of maxillary anterior teeth toward the mandibular condyle in the glenoid fossa, thereby minimizing their destabilizing effect [17]. This focused; largely vertical force application pattern provides a more physiologically desirable loading pattern to the TMJ structures, i.e., the articular disc, retrodiscal tissues and fibrocartilaginous covering of the condylar head [18]. The much lower Helkimo dysfunction scores found in the lingualized occlusion group are consistent with prior studies reporting a decreased occurrence of masticatory muscle hyperactivity in this type of occlusal position [19]. Over activity of the muscles, especially the lateral pterygoid and masseter muscles, has been identified as a cause of TMJ dysfunction, due to distortion of condylar loading patterns and elevated intra-articular pressure [20]. The reduced lateral elements of force in lingualized occlusion may be why parafunctional muscle activity is compromised, thereby decreasing the mechanical load on TMJ structures. The VAS pain scores showed a significant and progressive decrease across all groups over time, partly due to neuromuscular adaptation to the new prostheses. Nevertheless, the decrease in pain in the lingualized occlusion group is much more pronounced, suggesting that the biomechanical benefits of this scheme extend beyond mere adaptation. It has been found that incorrect occlusal loading may activate nociceptive nerve endings in the TMJ capsule and retrodiscal tissues, resulting in pain and an inflammatory response [21]. The force-directing properties of lingualized occlusion might inhibit such nociceptive stimulation, ensuring that the condylar loads are more physiological. A TMJ click is higher in the monoplane occlusion group at six months and is clinically significant. Displacement of the disc that is usually accompanied by a click and reduction of the same indicates that the relationship between the condyles and the disc can be negatively influenced because of the application of the flat occlusal plane, such as those experienced with monoplane arrangements [22]. In the absence of cuspal guidance in lateral and protrusive movements, the mandible can take abnormal movement patterns that impose abnormal periods of stress on the articular disc and the attachments [23]. The experiment contradicts the conventional belief that removal of cuspal inclines with flat tooth would decrease TMJ strain, by reducing the amount of horizontal force. The CBCT examination of condylar positioning offered radiographic data of the clinical results. The almost concentric condylar alignment in the lingualized occlusion group features some of the best spatial relationships of the glenoid fossa, which are typically associated with healthy TMJ status [24]. Conversely, the tendency toward posterior displacement observed in the monoplane occlusion group raises questions about possible compression of highly vascular and innervated retrodiscal tissues, which may contribute to pain, inflammation and progressive joint dysfunction over the long term [25]. The bilateral balanced occlusion group also showed mediocre outcomes across most parameters. Although this scheme has always been regarded as the gold standard of complete denture occlusion, its requirement for precise tooth contacts at all excursive motions produces a complex pattern of force distribution that may not necessarily be healthy for the TMJ [26]. The inherent forces on the balancing side of this scheme induce medially directed forces on the working side and laterally directed forces on the balancing side, which are likely to cause asymmetric loading in the temporomandibular joints [27]. Moreover, the clinical problem of preserving ideal bilateral balance throughout life, as the denture base is subject to tissue adaptation, implies that the benefits of such a plan in theory may decrease with denture wear [28]. The positive change in maximum mouth opening across all groups is favorable, suggesting that prosthetic rehabilitation with any of the three occlusion schemes improves mandibular mobility. But the greater improvements of the lingualized occlusion group indicates that less muscle guarding and less pain are related to this scheme and that full restoration of the normal range of movement of the mandible is possible [29]. Pain or mechanical interference with the joint, causing limited anterolateral mouth opening, is often explained by protective splinting of the muscles of the mouth in response to ankle-brachial index dysfunction and is minimized by lingualized occlusion. The results of this research have significant clinical implications for prosthodontic practice, particularly in clinical decision-making. Although the selection of the occlusal scheme has long been influenced by the main factors of denture stability, masticatory ability and simplicity of production, the current findings may indicate that TMJ well-being should also be considered an important aspect of the scheme [30]. This is especially important to older edentulous patients who might already have subclinical aging-related TMJ changes and in whom any further biomechanical insult of having the wrong kind of prosthetic occlusion may trigger symptomatic dysfunction.

## Conclusion:

Lingualized occlusion showed superior TMJ outcomes, with lower Helkimo scores, reduced pain and joint sounds, improved condylar positioning and greater mouth opening at 6 months. Monoplane occlusion demonstrated the poorest results, including higher rates of clicking and condylar displacement. Thus, occlusal scheme selection strongly influences TMJ health, supporting lingualized occlusion as the preferred approach pending further validation.
